# *Drosophila* Smad2 degradation occurs independently of linker phosphorylations

**DOI:** 10.17912/micropub.biology.001153

**Published:** 2024-03-26

**Authors:** Kenny Castro, Volodia Muradyan, Pablo Flota, John Guanzon, Neil Poole, Hugo Urrutia, Edward Eivers

**Affiliations:** 1 Biological Sciences, California State University Los Angeles, Los Angeles, California, United States; 2 Division of Biology and Biological Engineering, California Institute of Technology, Pasadena, California, United States

## Abstract

TGF-β signals are important for proliferation, differentiation, and cell fate determination during embryonic development and tissue homeostasis in adults. *Drosophila* Activin/TGF-β signals are transduced intracellularly when its transcription factor dSmad2 (also called Smad on X or Smox) is C-terminally phosphorylated by pathway receptors. Recently, it has been shown that receptor-activated dSmad2 undergoes bulk degradation, however, the mechanism of how this occurs is unknown. Here we investigated if two putative linker phosphorylation sites are involved in dSmad2 degradation. We demonstrate that degradation of activated-dSmad2 occurs independently of threonine phosphorylation at linker sites 252 and 277. We also show that dSmad2 degradation is not carried out by cellular proteasomes.

**Figure 1. dSmad2 degradation occurs independently of linker phosphorylations:**
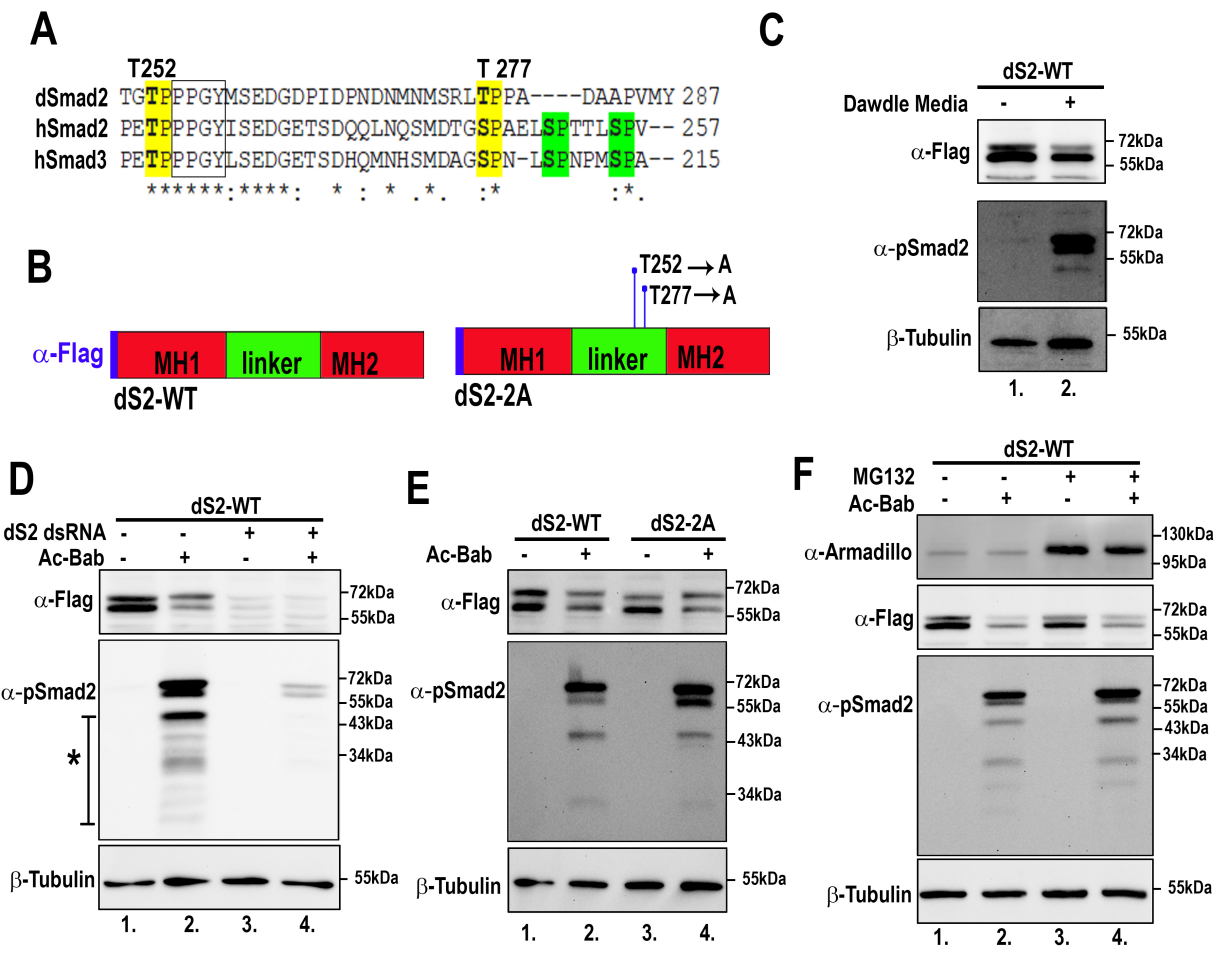
( **A** ) Partial alignment of the linker regions of dSmad2 and human Smads 2 and 3. Conserved linker phosphorylation sites highlighted in yellow, while two additional human linker phosphorylation sites, highlighted in green, are absent in dSmad2. ( **B** ) Schematic diagram showing the general structure of dSmad2 and locations of mutated linker threonines. ( **C** ) Dawdle treatment of S2R+ results in C-terminal phosphorylation of dSmad2 and a decrease in total dSmad2 protein levels ( **D** ) Phosphorylation of dSmad2 by Ac-Bab causes bulk degradation of dSmad2. ( **E** ) Linker phosphorylation sites do not seem to be involved in the bulk degradation of dSmad2. Quantification of the Flag-dSmad2 bands using ImageJ showed a 50% drop in total Flag-dSmad2 levels when comparing lanes 1 to 2 and lanes 3 to 4 when normalized to β-Tubulin **(F)** Treatment of S2R+ cells with the proteasomal inhibitor MG132 does not result in dS2-WT stabilization. Quantification of the Flag-dSmad2 bands using ImageJ showed a 75% drop in total Flag-dSmad2 levels when comparing lanes 1 to 2 and a 62% drop in total Flag-dSmad2 levels when comparing lanes 3 to 4, when normalized to β-Tubulin.

## Description

The Transforming Growth Factor β (TGF-β) superfamily can be subdivided into the Bone Morphogenetic Protein (BMP) and the TGF-β/Activin signaling pathways (Massague 1998; Shi & Massague, 2003). In responding cells, each pathway utilizes a different class of transcription factors known as receptor-regulated Smads (R-Smads) to transduce their intracellular signals. The BMP pathway recruits Smad1 (Mad in *Drosophila* ), while the TGF-β/Activin pathway recruits Smad2/3 (dSmad2 in *Drosophila* ) (Massague 1998; Shi & Massague, 2003; Upadhyay et al., 2017). To activate each pathway, an R-Smad binds to a ligand-activated serine/threonine kinase receptor complex, which in turn phosphorylates Smad proteins at two C-terminal serine residues (SXS). Activated R-Smads then interact with a co-Smad, Smad4 (Medea in *Drosophila* ) to form a signaling complex, which enters and accumulates in the nucleus to activate or repress target genes (Massague 1998; Shi & Massague, 2003; Upadhyay et al., 2017). Activated R-Smads are highly regulated and one way to terminate their signals is by initiating a cascade of cellular events resulting in their degradation. This is achieved by phosphorylation of R-Smads in their central linker region at serine/threonine-proline motifs by a number of different kinases, which include; Cyclin Dependent Kinase 8/9 (CDK8/9), Extracellular signal-regulated kinase (ERK), P38 and Jnk. This is then followed by ubiquitin ligase binding to a nearby PY motif, polyubiquitination, and finally degradation by cellular proteasomes. (Kretzschmar et al., 1997; Zhu et al., 1999; Lo & Massague, 1999; Pera et al., 2003; Aubin et al., 2004; Kuroda et al., 2005; Sapkota et al., 2007; Fuentealba et al., 2007; Eivers et al., 2009; Gao et al., 2009; Aleman et al., 2014) .

The *Drosophila* TGF-β/Activin signaling pathway has four ligands: dActivin (dAct), Dawdle (Daw), Maverick (Mav) and Myoglianin (Myg), which bind to transmembrane receptor complexes of Baboon and either Punt or Wit, leading to C-terminal phosphorylation of dSmad2 (Henderson & Andrew, 1998; Brummel et al., 1999; Lo & Frasch, 1999; Nguyen et al., 2000; Lee-Hoeflich et al., 2005; Serpe & O’Connor, 2006; Parker at al., 2006; Gesualdi & Haerry, 2007; Zhu et al., 2008; Peterson et al., 2012). Biochemically, it has been demonstrated that C-terminal phosphorylation of dSmad2 by the receptor Baboon, leads to TGF-β/Activin pathway activation, followed by dSmad2 bulk degradation in vivo (Peterson et al., 2012) . To date, no mechanism has been described as to how dSmad2 bulk degradation occurs in *Drosophila* . Comparing the amino acid sequence of dSmad2 to human Smad2/3, we identified two threonine-proline motifs adjacent to a conserved PY motif in the linker domain of dSmad2 ( Figure 1A ). In this study, we tested if linker threonines 252 and 277 and/or cellular proteasomes play a role in dSmad2 degradation.

Using *Drosophila* cDNA, we PCR amplified full-length dSmad2 (dS2-WT) and inserted it into a pUAST expression vector, our forward primer contained the flag-tag sequence to allow for antibody detection of total Flag-dSmad2 protein on western blots ( Figure 1B ). In addition to dS2-WT, we generated a mutant dSmad2 construct, dS2-2A, which had two conserved linker phosphorylation sites, threonines 252 and 277, mutated into non-phosphorylatable alanines ( Figure 1A -B). These conserved linker sites have previously been shown to be involved in human Smad3 turnover (Gao et al., 2009) . First, we tested our new dS2-WT construct to confirm bulk degradation occurs in response to phosphorylation of its C-terminal domain. We transfected *Drosophila* S2R+ cells with dS2-WT and three days post-transfection we treated cells either with or without Dawdle-conditioned media for 8 hours before lysing cells. Western blot analysis of cell lysates revealed that in Dawdle-treated cells, dSmad2 was C-terminally phosphorylated and total levels of flag-tagged dS2-WT were slightly decreased when compared to non-Dawdle-treated cells ( Figure 1C, compare lanes 1 and 2). Next, we transfected S2R+ cells with dS2-WT either with or without a constitutively activated form of Baboon (Ac-Bab). Again, when dS2-WT was phosphorylated, we saw a decrease in total flag-tagged dS2-WT protein levels when compared to cells that were not co-transfected with Ac-Bab ( Figure 1D, compare lanes 1 and 2). Both these findings confirmed that when dSmad2 is activated in cells (using an Ac-Bab or Dawdle conditioned media), it appears to undergo bulk degradation ( Figure 1C and D ). We also noticed that when dSmad2 was C-terminally phosphorylated by pathway receptors, the pSmad2 antibody detected a series of lower bands below the full-length dSmad2 on western blots ( Figure 1D, α-pSmad2, line/asterisk). Using dSmad2 RNAi we found that these lower bands were lost alongside full-length dSmad2 ( Figure 1, compare lanes 2 and 4), suggesting that these lower bands may be partial degradation products of phosphorylated dSmad2. Next, we investigated if mutation of threonines 252 and 277 into non-phosphorylatable alanines could affect the bulk degradation of dSmad2 proteins. We transfected S2R+ cells with dS2-WT with or without Ac-Bab and another set of cells with dS2-2A with or without Ac-Bab. Dual mutation of threonines 252 and 277 into non-phosphorylatable alanines resulted in no detectable stabilization of C-terminally activated dS2-2A proteins when compared to dS2-WT and we also did not see any change in intensity of the lower bands picked up by the pSmad2 antibody ( Figure 1 E, compare lanes 2 and 4). Finally, we tested if dSmad2 bulk degradation is carried out by cellular proteasomes, as has been described for other Smad proteins (Sapkota et al., 2007; Fuentealba et al., 2007; Eivers et al., 2009; Gao et al., 2009) . dS2-WT was transfected in S2R+ cells with and without Ac-Bab, 6 hours before lysing the cells, two wells were treated with the proteasomal inhibitor MG132, while two other wells were treated with DMSO (solvent used to resuspend MG132). No stabilization of activated dSmad2 and no change in the lower pSmad2 bands were found in cells treated with the proteasomal inhibitor MG132 ( Figure 1 F, compare lanes 2 and 4). As a positive control for this experiment, we measured total levels of Armadillo, which is known to be degraded by proteasomes (Swarup et al., 2011) , Armadillo was found to be stabilized in the presence of MG132. In conclusion, we found that the two putative dSmad2 linker phosphorylations investigated here do not seem to be involved in the bulk degradation of dSmad2 and activated dSmad2 proteins were not degraded by cellular proteasomes under these experimental conditions.

## Methods

**Plasmids and RNAi**

The FlyBase ID for dSmad2 (Smox) is FBgn0025800. The full-length dSmad2 coding sequence was PCR amplified using *Drosophila* cDNA from S2R+ cells, the forward dSmad2 PCR primer also contained the Flag sequence. Flag-tagged, dS2-WT and dS2-2A were cloned into the *Drosophila* expression vector pUAST. Activated – Baboon plasmid was a gift from M.B. O'Connor. dSmad2 RNAi was generated using primers described in Peterson et al., 2012. RNAi treatment of S2R+ cells was followed as described in Clemens et al., 2000.

**Cell culture and Immunoblotting**

*Drosophila* S2R+ cells were cultured using standard protocols. The Effectene kit (Qiagen) was used to transfect plasmid DNAs into S2R+ cells, using the manufacturer's instructions. A Dawdle construct was transfected into S2R+ cells and 3 days post-transfection conditioned media was harvested. S2R+ cells were treated with 0.22µM of MG132 for 6 hours before lysing. All cell samples were lysed in RIPA buffer containing phosphatase and protease inhibitors. All western blotting was carried out using standard protocols. Primary antibodies were used at the following concentrations, pSmad2 (Cell Signaling), 1:1000; anti-Flag (Sigma), 1:2000, anti-β-Tubulin (E7-c, Hybridoma bank), 1:1000; anti-Armadillo (N2 7A1, Hybridoma Bank), 1:1000. Secondary antibodies (Thermo Scientific) anti-mouse, and anti-rabbit 1:1000. Western blots were imaged using a versa doc imaging system 5000MP (Bio-Rad).
